# Intrahepatic Cholestasis of Pregnancy: Diagnosis, Management, and Future Directions—A Review of the Literature

**DOI:** 10.3390/diagnostics15162002

**Published:** 2025-08-10

**Authors:** Kamil Jasak, Wanda Gajzlerska-Majewska, Zoulikha Jabiry-Zieniewicz, Ewelina Litwińska-Korcz, Magdalena Litwińska, Artur Ludwin, Monika Szpotańska-Sikorska

**Affiliations:** Department of Obstetrics and Gynecology, Medical University of Warsaw, 02-091 Warsaw, Poland; kamil.jasak@wum.edu.pl (K.J.); wanda.gajzlerska-majewska@wum.edu.pl (W.G.-M.); zzieniewicz@gmail.com (Z.J.-Z.); ewelina.litwinska-korcz@wum.edu.pl (E.L.-K.); magdalena.litwinska@wum.edu.pl (M.L.); artur.ludwin@wum.edu.pl (A.L.)

**Keywords:** intrahepatic cholestasis of pregnancy, pregnancy pathology, bile acids, ursodeoxycholic acid, perinatology

## Abstract

Intrahepatic cholestasis of pregnancy (ICP) is the most common liver disorder specific to pregnancy, typically presenting in the third trimester. It is characterized by pruritus, elevated serum bile acids, and abnormal liver function tests. While maternal symptoms resolve postpartum, ICP poses significant risks to fetal health, including spontaneous preterm labor, meconium-stained amniotic fluid, and stillbirth. This review aims to synthesize current knowledge on the pathogenesis, diagnosis, and management and highlight emerging research and possible therapy directions in ICP. A comprehensive review of recent literature was conducted, focusing on molecular mechanisms, clinical management guidelines, fetal outcomes, and novel therapeutics under investigation. Ursodeoxycholic acid (UDCA) remains the primary pharmacologic treatment of intrahepatic cholestasis of pregnancy; however, its effect on perinatal outcomes is debated. Investigational therapies—including Volixibat, FXR agonists, 4-phenylbutyrate, and NorUDCA—are under exploration. These emerging therapies hold the potential to improve both maternal symptoms and perinatal outcomes by addressing the underlying pathophysiology of ICP more effectively than current standard treatment. Additionally, emerging biomarkers and machine-learning tools hold promise for improved diagnosis and personalized care. ICP continues to pose diagnostic and therapeutic challenges. While maternal outcomes are generally favorable, optimizing fetal safety requires timely diagnosis, stratified risk assessment, and evidence-based delivery planning. Future research should prioritize identifying predictive biomarkers, refining treatment algorithms, and assessing long-term outcomes for both mothers and offspring. Special attention should also be given to the investigation of novel therapeutic targets.

## 1. Introduction

Intrahepatic cholestasis of pregnancy (ICP) is a liver disorder unique to pregnancy, characterized by reversible cholestasis. This pathology was first described in 1883 by Ahlfeld as recurrent jaundice in pregnancy that resolved after the delivery. Historically, ICP has been referred to by several names, including jaundice of pregnancy, recurrent pregnancy-associated jaundice, idiopathic jaundice of pregnancy, obstetric hepatosis, gestational hepatosis, and obstetric cholestasis [1]. Pruritus is the primary symptom of ICP, usually involving the palms and soles [2]. Intrahepatic cholestasis of pregnancy typically presents with maternal pruritus with raised serum bile acid (BA) concentrations [3]. The maternal prognosis is generally favorable [3]. The clinical and biochemical abnormalities usually resolve within two to four weeks postpartum; however, ICP has a high recurrence rate in subsequent pregnancies [4]. To ensure optimal postpartum care for women with ICP, liver function tests should be performed. This is important because, in some cases, persistent laboratory abnormalities in the postpartum period may indicate chronic liver dysfunction [5]. Although ICP predominantly manifests in the late second or third trimester, early-onset cases, including as early as five weeks’ gestation in spontaneous pregnancies, have been documented [6].

The etiology of ICP is multifactorial and incompletely understood. It is thought to involve the cholestatic effects of reproductive hormones in genetically predisposed women [7]. The pathogenesis of the disease involves impaired transport of bile constituents from the basolateral membrane of the hepatocyte to its canalicular pole. Altered maternal lipid and bile acid metabolism disrupts hepatic bile transport, resulting in elevated BA levels in the serum [8]. Pregnancies with maternal bile acid levels under 40 μmol/L are rarely associated with adverse outcomes [2]. Elevated BA levels over 40 μmol/L are linked to poor obstetric and neonatal outcomes, such as preterm birth, low birthweight, and meconium-stained amniotic fluid, likely due to intrauterine hypoxia and ischemia. A concentration exceeding 100 μmol/L is associated with the highest risk of stillbirth [9].

Intrahepatic cholestasis of pregnancy is commonly treated with ursodeoxycholic acid. This treatment is effective in improving maternal symptoms and reducing bile acid levels in some women with ICP. However, data regarding its overall efficacy remain debatable [7,10,11].

Perinatal care for pregnant women with intrahepatic cholestasis of pregnancy represents a significant challenge for gynecologists and obstetricians due to the insidious and often unpredictable course of the disease.

## 2. Material and Methods

This study represents a narrative review of the literature. Its purpose is to provide an updated overview of intrahepatic cholestasis of pregnancy (ICP). The focus is on limitations in current management options and recent advancements with potential for clinical application. A literature search was conducted by three practicing clinicians (K.J., W.G.-M., and M.S.-S.) using the PubMed database (National Center for Biotechnology Information, Bethesda, MD, USA). We included original research articles, clinical guidelines, and review articles concerning intrahepatic cholestasis of pregnancy. Literature was not chosen within a formal meta-analytical or systematic framework. Publications were selected to emphasize key aspects of ICP. The focus was on novel findings and studies published from 2020 onward. Selected earlier landmark studies were also included to provide background. In addition to the database search, we applied a snowballing approach, reviewing reference lists of key articles to ensure comprehensive coverage of the relevant literature. The selected studies were analyzed and synthesized to identify major themes and provide an updated perspective on Intrahepatic Cholestasis of Pregnancy. References were managed using EndNote, version 20.3 (Clarivate, Philadelphia, PA, USA).

## 3. Epidemiology

The prevalence of ICP varies based on geography, ethnicity, and socioeconomic status, and diagnostic criteria. Globally, its incidence is estimated at 2.9% of pregnancies. The incidence of ICP was reported as 0.8% in the United States [12], 3.1% in Pakistan [13], and 6.1% in China [14]. Meta-analyses data indicate that the Southeast Asian region has the highest prevalence at 4.7%, while the Eastern Mediterranean region shows the lowest at 1.6% [15]. Developing nations and low- to middle-income countries report higher incidence rates compared to high-income regions. Clinically significant ICP with BA > 40 μmol/L is estimated to affect approximately 1 in 1000 pregnancies [1]. Risk factors include pre-pregnancy underweight or obesity, inadequate gestational weight gain, and multiple gestations. Other factors are maternal age under 25 or 35 years and above, multiparity, and a history of inflammatory bowel disease (IBD) [14,16,17]. HBV infection is another significant risk factor for developing ICP [18,19,20]. Additional risk factors for severe ICP include a history of ICP, pregestational diabetes, prior cholecystectomy, and tobacco use [3]. In vitro fertilization (IVF) may also be a risk factor of ICP. Some studies have reported a higher rate of ICP in singleton pregnancies conceived via IVF compared to those conceived spontaneously [21,22]. IVF pregnancies have also been associated with higher maternal BA levels [23]. Furthermore, IVF may independently increase the risk of adverse perinatal outcomes in women with ICP [24].

## 4. Etiology and Pathogenesis

The pathophysiology of ICP is still unclear. Pathogenesis likely results from genetic, hormonal, and environmental factors. Elevated concentrations of reproductive hormones during pregnancy likely reveal symptoms and biochemical features of ICP in genetically susceptible individuals. These hormones may also predispose cholestasis in women with previously asymptomatic liver disease [25].

### 4.1. Genetics

Familial clustering supports a genetic basis, with first-degree female relatives of affected women at higher risk [26,27]. The recurrence rate in subsequent pregnancies is approximately 60% [28]. Mutations in the ATP-binding cassette (ABC) transporter family, particularly in the following genes: ABCB4, ABCB11, and ATP8B1, have been associated with ICP. ABCB4 encodes a phosphatidylcholine transporter critical for micelle formation in bile, MDR3. MDR3 dysfunction leads to bile acid accumulation and hepatocellular injury [29]. ABCB11 encodes bile salt export pump (BSEP), the major transporter responsible for moving bile acids from hepatocytes into bile canaliculi. ATP8B1 encodes a *p*-type ATPase that maintains phospholipid asymmetry in hepatocyte membranes, essential for membrane stability and bile acid resistance. Mutations in ABCB4 and ABCB11 genes are associated with severe early-onset ICP [30,31]. FXR is a nuclear receptor that regulates BA synthesis, secretion, and reabsorption. Heterozygous mutations in the NR1H4 variant have been identified in some women with severe or recurrent ICP [32].

### 4.2. Hormones

During pregnancy, both estrogen and progesterone levels rise significantly, peaking in the third trimester. Estrogen contributes to cholestasis by reducing BSEP activity in hepatocytes and desensitization of the FXR by 17β-estradiol. This mechanism is supported by the association between oral contraceptive use and cholestasis [29]. Furthermore, intrahepatic cholestasis has been observed in women treated with oral contraceptives [33]. It has also been reported in ovarian hyperstimulation syndrome [34]. Progesterone plays a role by impairing BSEP function through progesterone sulfate. It also affects the FXR pathway. Elevated progesterone levels, particularly in multiple pregnancies or with exogenous administration, may impair gallbladder motility. This impairment increases the risk of cholestasis [35,36].

### 4.3. Environment

Environmental factors such as air pollution and low sunlight exposure during preconception are emerging contributors to ICP risk [37,38]. Some research indicates a potential role for gut microbiota dysbiosis in the pathogenesis of ICP. Gut microbiota play a significant role in bile acid metabolism through enzymatic processes such as deconjugation and dehydroxylation. These reactions convert primary bile acids into secondary bile acids. Such changes alter the composition of the bile acid pool and affect signaling through receptors like FXR and TGR5. Disrupted intestinal flora may alter bile acid composition and hepatic metabolism, thereby contributing to cholestasis in genetically susceptible women [39,40].

### 4.4. Pathogenesis

The pathogenesis of ICP involves a disturbance in the transport of bile components from the basolateral part of the hepatocyte to its canalicular (bile-facing) pole. As a result of impaired transport within the hepatocyte, intracellular bile flow is disrupted, leading to increased permeability of intercellular junctions. Consequently, this causes abnormal circulation of BA and, as a result, an increase in their concentration in the peripheral blood. Increased maternal BA levels impact placental function at the molecular level. Mitochondrial damage and autophagy activation in trophoblasts contribute to placental insufficiency and adverse perinatal outcomes [41,42]. Due to impaired bile flow, a vitamin K deficiency may be observed in women with ICP, potentially increasing the risk of post-partum hemorrhage [43,44]. Probably inflammatory mediators are also involved in the development of ICP. Therefore, ICP is often accompanied by varying degrees of abnormalities in inflammatory factors; however, the specific role of inflammation in the pathogenesis of ICP is not known [45]. Concerning the maternal symptoms, the cause of pruritus in ICP is not fully understood. Probably, BAs are only partly responsible for pruritus. The lysophosphatidic acid (LPA) and sulfated progesterone metabolites were presented as potential pruritogens which may directly stimulate pruritus pathways. In ICP, elevated autotaxin activity increases LPA production, while genetic variants in PNPLA3 may impair its breakdown, enhancing susceptibility. Sulfated progesterone metabolites, particularly 5β-pregnan-3α-20α-diol-sulfate (PM3S), correlate with itch severity and activate the TGR5 receptor [26].

## 5. Diagnosis

Pruritus, the primary symptom of ICP, usually occurs in the late second or third trimester. Most typically the palms of the hands and soles of the feet are affected, but it may emerge on all body parts. There is the tendency that pruritus exacerbates during the night and may lead to insomnia, mood disorders, and depression [46]. The severity of pruritus is assessed with the visual analog scale (VAS); however, its severity does not correlate with the maternal bile acid serum level [47]. In 17–75% of cases of ICP, jaundice may occur. Usually, jaundice emerges 1–4 weeks after the onset of pruritus [46,48]. Contrary to pruritus, jaundice usually does not deteriorate with advancing gestational age [49]. Rash is not typically associated with ICP, but affected women may develop dermatitis artefacta secondary to scratching [8]. Typical symptoms of cholestasis, such as anorexia, malaise, abdominal pain, pale stools, dark urine, and steatorrhea, may also be present but they usually do not constitute elements of the clinical manifestation. [50] Laboratory abnormalities in ICP concern primarily elevated total serum bile acid concentration. A threshold of BA > 10 μmol/L is commonly used in studies and clinical practice [51], although according to the guideline from the Royal College of Obstetricians and Gynecologists (RCOG) diagnosis of ICP requires an elevated BA level of 19 μmol/L or more in pregnancy [52]. The recommendations from the Society for Maternal-Fetal Medicine suggest the diagnosis of ICP with any increase in BA levels above the upper limit of normal in a pregnant woman with pruritus. The highest concentration of BA occurs after a meal. Non-fasting BA testing improves sensitivity for high-risk ICP detection, though fasting levels offer higher specificity [53]. Measuring bile acids one hour after eating reduces the risk of incorrectly classifying a patient into the incorrect ICP severity group. After diagnosis, the pregnant woman should have her bile acid levels measured once a week until the delivery. Other laboratory findings may contain an increase in the activity of aminotransferases, particularly alanine aminotransferase (ALT). Aminotransferases activity, often 2–25 times the upper limit of normal is present in 60–85% of patients. [54,55]. Aminotransferases concentration does not correlate with the severity of ICP and therefore has no predictive value for ICP-related complications [9]. However, single studies suggested that the aspartate aminotransferase ratio with platelets may be increased during pregnancy, even in the first trimester in women with ICP. Therefore, it may be a valuable and cheap predictor of ICP [56,57]. Bilirubin is typically mildly elevated (<5 mg/dL). Its concentration also does not correlate with the severity of ICP [9]. Alkaline phosphatase may rise up to 4-fold, and GGT is elevated in about 30% of patients [58]. ICP is confirmed retrospectively when serum liver tests and pruritus completely normalize within 3 months after delivery [25].

## 6. Management

The aim of currently available pharmacotherapy is to reduce adverse pregnancy outcomes while improving maternal pruritus and biochemical markers of liver injury. The goal of obstetric management is to prevent the most serious complication—stillbirth—through timely induction of labor.

### 6.1. First-Line Therapy

Ursodeoxycholic acid (UDCA) at dose of 10–15 mg/kg/day divided into two or three dosages is the primary pharmacologic therapy. UDCA is one of the natural bile acids present in the human body in small amounts. Its therapeutic effect is based on pleiotropic effects on liver metabolism. It stimulates bile acid secretion through upregulation of hepatic enzymes and bile acid transporters. Also, it alters post-transcriptional mechanisms, leading to stimulation of impaired hepatocellular secretion. UDCA is thought to stabilize cell membranes and provide a protective role against the toxic effects of biliary acids. It also has immunomodulatory effects by reducing pro-inflammatory mechanisms, including blocking immunophagocytosis [10]. Cochrane database analysis showed that UDCA treatment in ICP is clearly associated only with partial reduction in pruritus. UDCA appeared to be beneficial in reducing preterm birth, but the evidence quality was moderate to low. Importantly, the effect of therapy was unclear for other adverse fetal outcomes [59]. No other drugs effective in ICP treatment were found in this meta-analysis. Ovadia et al.’s meta-analysis [11] did not show a significant reduction in stillbirth with ursodeoxycholic acid treatment and had no effect on the prevalence of the composite outcome (stillbirth, preterm birth, and neonatal unit admission).

### 6.2. Alternative Options

For women with ICP not responding to UDCA, other therapeutic options are considered. This therapy involves medications, such as rifampicin, cholestyramine, and S-adenosyl-L-methionine [60]. Rifampicin increases bile acid detoxification and excretion and can be an added to UDCA. Cholestyramine is an anion exchange resin that decreases the ileal absorption of bile salts, thereby increasing their fecal excretion. S-adenosyl-L-methionine is a naturally occurring compound in the body, which increases methylation and biliary excretion of hormones and improves liver detoxification pathways. Also, antihistamine drugs are used in ICP to alleviate pruritus [61].

### 6.3. Lack of Evidence for Other Agents

According to Cochrane meta-analysis [59], there is no evidence that S-adenosylmethionine, guar gum, activated charcoal, dexamethasone, cholestyramine, or Chinese herbal medicines alone or in combination are effective in treating women with cholestasis of pregnancy. The efficacy of topical emollients was not assessed in clinical trials.

### 6.4. Ongoing Clinical Trials

In 2025, the clinical trial TURRIFIC, comparing rifampicin vs. UDCA in reducing pruritus in women with severe early-onset ICP, was completed. The results of this study will provide high-quality evidence for the use of rifampicin in severe ICP for the first time [62].

### 6.5. Labor Induction in ICP

ICP is associated with a significantly increased risk of both spontaneous and iatrogenic preterm delivery. In women with ICP, preterm delivery occurs in 32.5% of pregnancies [63]. Three ICP severity groups are usually distinguished based on bile acid (BA) concentration: mild (BA 10–39 μmol/L), moderate (BA 40–99 μmol/L), and severe (BA ≥ 100 μmol/L) [64]. Elevated BA levels (especially > 40 μmol/L) are associated with low birthweight, meconium-stained amniotic fluid due to intrauterine ischemia, and postpartum hemorrhage. The risk of adverse fetal outcomes increases with higher maternal BA levels and advancing gestation [1]. The risk of stillbirth increases in women with total BA concentrations ≥ 100 μmol/L at any point during pregnancy [65]. This is due to possible fetal arrhythmia and placental vasoconstriction associated with increased BA concentration [66,67]. Elevated fetal and maternal serum BA concentrations in untreated ICP are associated with an abnormal fetal cardiac phenotype characterized by increased NT-proBNP concentration, elongated PR interval, and heart rate variability [68]. The highest bile acid concentration recorded throughout the whole pregnancy is the best predictor of stillbirth, with no association between timings of bile acid measurement [11]. To date, no other reliable predictors of sudden fetal death have been identified. To reduce the risk of stillbirth, induction of labor is recommended. The BA level is considered the most important factor in determining the timing of labor induction. Bile acids do not need to be tested in a fasting state, and the highest recorded bile acid in pregnancy should be taken into consideration. After diagnosis, delivery timing requires balancing the risk of stillbirth with the potential for neonatal morbidity at early gestational age [69]. The guidelines of the Society for Maternal–Fetal Medicine and the American College of Obstetricians and Gynecologists recommend delivery at 36 weeks in severe cholestasis (BA ≥ 100 μmol/L) or at 34–36 weeks in severe cholestasis complicated with unremitting maternal pruritus, a prior history of a stillbirth before 36 weeks’ gestation due to ICP, or preexisting or acute hepatic disease with worsening liver function. For the ICP with BA concentration < 100 μmol/L, delivery between 36 and 39 weeks of gestation stratified by bile acid level should be proposed. Other societies propose different timing of labor induction in ICP. According to the Royal College of Obstetricians and Gynecologists, in women with severe ICP (peak BA ≥ 100 micromol/L) birth should be planned at 35–36 weeks’ gestation; in moderate ICP (peak BA 40–99 micromol/L) at 38–39 weeks’ gestation; and in mild ICP (peak BA 19–39 micromol/L) with no other risk factors birth should be planned by 40 weeks of gestation. More strict recommendations are presented by the International Federation of Gynecology and Obstetrics (FIGO). Based on FIGO guideline optimal timing for delivery is 35–36 weeks’ gestation, 36–39 weeks’ gestation (closer to 36 weeks) and 37–39 weeks’ gestation for severe, moderate and mild ICP, respectively. The summarized recommendations are presented in Table 1.

### 6.6. Broad Perspective on ICP

The retrospective data suggest that women with a multisymptomatic course of ICP may be at higher risk of perinatal complications, including a higher risk of coexisting gestational diabetes mellitus (GDM), as well as increased risks of preterm birth and neonatal intensive care unit admissions [72,73]. Women with a history of ICP are at higher risk of developing cholelithiasis, cholecystitis, pancreatic disfunction, and hypothyroidism compared to physiological pregnancies [74]. Women with this diagnosis should be carefully observed for other comorbidities. Prenatal influence of BA may also affect in further life. There is a possible association that children born to women with ICP may be at higher risk of developing conditions such as diabetes, obesity, and dyslipidemia [75]. More long-term observation is required to confirm the association.

## 7. Discussion and Future Directions

### 7.1. Fetal Monitoring and Prognostic Factors

In some of the aspects of pathophysiology and its influence on maternal and especially perinatal outcome, ICP still remains a puzzle. One of the crucial questions is how to avoid the most serious complication—the sudden intrauterine death. Fetal loss may be preceded by normal CTG and/or normal fetal movements. Majority of ICP pregnancies have a good biophysical profile [76]. Nevertheless, routine fetal monitoring based on cardiotocography and fetal ultrasound scans should be provided [77]. A promising direction in fetal monitoring in ICP may become fetal echocardiography. The TEI Index, or Myocardial Performance Index, is based on measuring both systolic and diastolic time intervals to assess the global cardiac dysfunction. A case-control study showed an increased TEI index in fetuses of women with ICP; moreover, the TEI index was significantly correlated with bile acid levels [78,79,80,81]. Some studies already showed an association between a high left ventricle TEI index and adverse perinatal outcome in ICP [82,83]. Data are still limited and unstandardized. It is important to note that the TEI index is not currently part of routine clinical screening and remains an investigational tool requiring further validation through larger prospective studies. A future multicenter prospective study may clarify the prognostic utility of the TEI index in ICP.

Several studies have focused on serum BA profiles in women with ICP. In physiological pregnancy, the level of chenodeoxycholic acid (CDCA) is similar to the level of cholic acid (CA). On the contrary, in women with ICP, CA levels are higher with a relative reduction in the proportion of the CDCA. The effect of UDCA therapy is normalizing the maternal CA:CDCA ratio [84]. The profile of BA in women with ICP may become a useful tool to increase the diagnostic precision of adverse pregnancy outcomes. As there is a lack of prognostic and predictive factors, maternal serum or urinary bile acid profiles may help to predict the maternal and neonatal outcomes in ICP [85,86].

Recent studies raised the issue of easily accessible and cheap calculated inflammatory biomarkers that might be an additional diagnostic tool for ICP [87,88,89]. These include the neutrophil-to-lymphocyte ratio (NLR) and the Systemic Immune–Inflammation Index (SII). The mean NLR is elevated in women with ICP compared to the control; moreover, there is also a significant correlation between fasting BA and NLR and the severity of the cholestasis [90]. While taking platelet counts (PLT) with neutrophil and lymphocyte counts, the SII may be calculated. The SII can be used to assess systemic inflammation. Elevated SII values in ICP support the evidence for the inflammatory properties of ICP but do not aid in diagnosing and determining its severity [91]. While SII and NLR can differentiate between women with and without ICP with moderate accuracy, they should be used in conjunction with traditional biomarkers and probably will not become a breakthrough in ICP diagnosis.

### 7.2. Pharmacotherapy

Despite being widely used, there is insufficient data on UDCA effectiveness in therapy of ICP. The data concerning UDCA effectiveness are inconsistent. In the multicenter trial (PITCHES) conducted in the UK on a group of 605 pregnant women with ICP, UDCA the therapy was compared with placebo. The primary perinatal outcome (perinatal death, preterm, delivery or neonatal unit admission for at least 4 h) was observed in 74 (23.0%) infants in the UDCA group compared with 85 (26.7%) infants in the placebo group (aRR 0.85, 95% CI 0.62 to 1.15; *p* = 0.279). The results showed that UDCA was statistically not effective in improving primary perinatal outcomes. Regarding maternal outcomes, a significant difference between the groups on post-randomization maternal itch score was found, which was lower in the UDCA group than in the placebo group (MD −5.7 mm, 95% CI −9.7 to −1.7 mm; *p* = 0.005). Chappel et al.’s study confirmed that there is no significant difference in most adverse perinatal outcomes between treatment with ursodeoxycholic acid and placebo. They concluded that only some subgroups of women with ICP, which have not yet been identified, could respond to UDCA. Systematic review and meta-analysis conducted by Walker et al. [59] confirmed the tendency to observe a decrease in pruritus after UDCA introduction in analyzed studies; however, the data were heterogenic. In this study, assessment for adverse fetal outcomes was unclear due to very low-certainty evidence. On the contrary, Ovadia et al.’s systematic review and meta-analysis [11] when considering only randomized clinical trials, found an association with a reduction in stillbirth in combination with preterm birth comparing therapy with UDCA vs. without UDCA (aOR 0.51, 95% CI 0.33 to 0.78; *p* = 0.002) providing evidence for the clinical benefit of UDCA treatment in ICP.

Moreover, Kong et al. in their meta-analysis, showed that UDCA is safe and improves both maternal and fetal outcomes in pregnancies complicated by ICP. UDCA was shown to decrease the number of premature births (RR, 0.56; 95% CI, 0.43–0.72; *p* < 0.001), fewer number of neonates in the intensive care unit (NICU) (RR, 0.55; 95% CI, 0.35–0.87; *p* < 0.05) and improvement in pruritus (RR 1.68; 95% CI, 1.12–2.52; *p* = 0.01) compared to placebo. However, high heterogeneity in pruritus improvement and normalization of biochemical findings in ICP treated with UDCA was observed. This may be associated with still unknown factors altering response to the treatment [59,65,92]. Identification of the group of patients with ICP who may benefit from UDCA therapy is crucial to provide adequate treatment.

Identifying predictive markers of response to UDCA in ICP has become an important area of investigation. Deniz et al. [93] study results suggest that increased levels of zonulin—a regulatory protein that increases the intestinal permeability, are associated with the severity of ICP and unresponsiveness to treatment with UDCA in ICP. Other studies also confirm the relationship between elevated zonulin levels and the severity of ICP and adverse pregnancy outcomes. [94,95]. Increased intestinal permeability may facilitate the entry of BA into the enterohepatic circulation causing elevated bile acid levels in the maternal bloodstream [96]. Gumus et al.’s study found that a high level of syndecan-1 and glypican-3- heparan sulfate proteoglycans, which have a role in cellular healing processes like cytokines and growth factors—was related to inadequate response to 7-day UDCA treatment [97].

Although UDCA is considered safe in pregnancy single studies on animal embryos have shown that UDCA concentrations above 100 mg/L cause cardiomyocyte injury in zebrafish. Adverse effects include increased mortality, slower heart rates, pericardial edema, and elevated oxidative stress in embryonic cardiomyocytes. These findings may suggest a need to optimize UDCA level monitoring in clinical practice [98].

Knowing the limitations of UDCA, there is growing necessity for alternative or adjunctive treatments. To improve maternal symptom control, reduce bile acid concentration more effectively and improve perinatal outcome novel agents targeting specific pathophysiological mechanisms are under investigation.

### 7.3. Potential Future Directions in Pharmacotherapy

An intestinal bile acid transporter (IBAT) inhibitor—Volixibat blocks BA reabsorption in the ileum, thereby increasing fecal BA excretion. In 2021–2022, a clinical trial assessing Volixibat’s safety, tolerability, and efficacy in pregnant women with elevated serum BA levels due to ICP was conducted. The data from the 4 participants showed that both bile acids and itching improved in ICP women treated with Volixibat, with a favorable safety profile. Although the results were promising, the study was terminated due to difficulties with patient enrollment [99].

Norursodeoxycholic acid (NorUDCA) is a side chain–shortened derivative of ursodeoxycholic acid that has shown enhanced choleretic, anti-inflammatory, and antifibrotic properties in cholestatic liver disease. Unlike UDCA, NorUDCA undergoes cholehepatic shunting, which amplifies its effect on bile flow and hepatocellular detoxification pathways. In clinical trials for primary sclerosing cholangitis, NorUDCA significantly reduced serum alkaline phosphatase and liver enzymes, indicating improved bile acid metabolism and hepatoprotection [100]. Although NorUDCA has not yet been tested in pregnant women, its favorable pharmacokinetic profile it as a promising candidate for future investigation in ICP therapy.

Farnesoid X receptor (FXR) agonists represent a promising class of investigational drugs for ICP. FXR is a nuclear receptor critical for maintaining bile acid homeostasis by regulating genes involved in bile acid synthesis, transport, and detoxification, notably suppressing CYP7A1, the rate-limiting enzyme in bile acid production [101]. Some of functional variants in FXR may predispose to ICP [32]. In animal models with a 17α-ethynylestradiol (E2)-induced cholestatic pregnancy, a highly selective FXR agonist, W450, was introduced, significantly decreasing BA levels. Moreover, W450 protected against the impairment of placentas, including severe intracellular edema and apoptosis of trophoblasts, and attenuated placental oxidative stress [102]. Similarly, in E2 induced gestational cholestasis in mice, another FXR agonist—obeticholic acid (OCA)—showed promising results. OCA administration in animal models of induced cholestasis was associated with decreased incidence of intrauterine growth restriction during cholestasis [103].

Not only direct modulation of FXR but also targeting the gut microbiota represents a promising approach in improving the therapeutic strategy in ICP. Intestinal microbiota participates in deconjugation of the secondary bile acids [104]. Women with ICP compared to uncomplicated pregnancies have different gut microbiota profiles [105,106]. Tang et al. [107] showed that the microbiomes of patients with ICP were primarily characterized by *B. fragilis*, and the presence of this bacteria was markedly increased in patients with severe ICP. *B. fragilis* was able to promote ICP by inhibiting FXR signaling via its bile salt hydrolase (BSA) activity to modulate bile acid metabolism. *B. fragilis*-mediated FXR signaling inhibition was responsible for excessive bile acid synthesis and interrupted hepatic bile excretion to ultimately promote the initiation of ICP. Moreover, they observed that transplantation of gut microbiota from patients with ICP was sufficient to promote an ICP phenotype in mice. In animal studies also a bacterium with a positive influence on ICP course has been identified- the *Roseburia intestinalis.* In fecal samples from ICP rats, the *Roseburia intestinalis* was significantly decreased. Moreover, the transplantation of *Roseburia intestinalis* significantly decreased blood bile acid levels in ICP rats improved bile acid synthesis and transport [108]. Not only the microbiota itself may vary a lot in ICP complicated pregnancies compared to physiological ones, but also short-chain fatty acids (SCFAs)- main metabolites of the gut microbiota. Ren et al. [109] analyzed SCFAs in maternal serum and umbilical cord blood, comparing ICP and healthy pregnancy groups. A significant positive correlation between maternal serum SCFAs and umbilical cord blood SCFAs was found and significant differences in maternal SCFAs were observed between the ICP and healthy pregnancy groups.

Another promising path is to find a new drug that is effective in ICP therapy. One such candidate is 4-phenylbutyric acid (4-PBA). 4-PBA is a chemically synthesized, low-molecular-weight aromatic fatty acid approved by the FDA for use in clinical trials in the treatment of urea cycle disorders in children. There is a gap for potential use of 4-PBA as new opportunity in ICP treatment, as it was showed to reduce bile acid levels and improve fetal outcomes on animal models [110].

Another theoretical point of interest in the therapy of ICP is an association between BACH1 transcriptional repressor and placental angiogenesis in ICP. BACH1 has been shown to enhance oxidative stress and downregulate angiogenesis. Animal studies showed that BACH1 is significantly elevated in placental vascular endothelial cells during ICP. Targeting BACH1 inhibition may represent a potential therapeutic strategy to improve perinatal outcome [111]. The potential future directions in pharmacotherapy are summarized in Table 2.

### 7.4. Artificial Intelligence in ICP

The rapid development in artificial intelligence will probably influence the diagnosis of ICP to provide adequate and early intervention. Ren et al. [112] identified factors (total bile acid, gamma-glutamyl transferase, multiple pregnancy, lymphocyte percentage, hematocrit, neutrophil percentage, prothrombin time, aspartate aminotransferase, red blood cell count, lymphocyte count, and platelet count) and developed machine learning models based on these factors on the group of 798 participants. The AUCs of the selected top five models ranged from 0.9509 to 0.9614. He et al. in their study [113] analyzed data from 1092 participants taking into consideration variables from blood morphology, biochemical results, urine routine results, and other ontogenic data. One of their models showed the accuracy (90.50%) for predicting ICP. Both studies demonstrated relatively good performance as a predictor of ICP. The biomarkers used in the models are easily accessible what makes the implementation in routine practice possible. Artificial intelligence may be useful in the case of limited resources when BA levels are unavailable. Asali et al. conducted the study where 336 pregnant women suffering from pruritus during the second and third trimesters used demographic, obstetrical, clinical, and laboratory data to predict bile acid levels ≥ 10 μmol/L [114]. The most accurate model AUC was 0.9. Using artificial intelligence models may improve identification of ICP, especially when bile acid testing is not available.

### 7.5. Summary

Intrahepatic cholestasis of pregnancy (ICP) remains a clinically significant yet incompletely understood hepatobiliary disorder of pregnancy. Despite its typically reversible postpartum course, ICP poses substantial risks for fetal complications, including spontaneous preterm birth, meconium-stained amniotic fluid, and stillbirth. Bile acid concentrations—especially levels ≥100 µmol/L—remain the most reliable biochemical marker of fetal risk. While ursodeoxycholic acid (UDCA) remains the first line of therapy, its benefits for fetal outcomes remain inconsistent, highlighting the urgent need for more targeted and personalized treatment strategies. Recent research has expanded our understanding of ICP pathogenesis, implicating genetic mutations, hormonal modulation of bile acid transport, and gut microbiota dysbiosis. Novel investigational therapies such as IBAT inhibitors, FXR agonists, 4-phenylbutyrate, and BACH1 inhibitors offer promising mechanistic targets but require validation through clinical trials. Similarly, metabolomic profiling and machine learning–based risk stratification tools are emerging as potential adjuncts for diagnosis, prognosis, and therapy selection.

Looking forward, future priorities in ICP research and clinical practice should include:

- Establishing predictive biomarkers of disease severity and treatment response (especially to UDCA)
- Conducting multicenter trials of emerging therapies with fetal outcome endpoints
- Clarifying the long-term metabolic effects of in utero bile acid exposure
- Standardizing global guidelines for diagnosis and induction timing

A multidisciplinary, evidence-based, and individualized approach to care—integrating obstetrics, hepatology, and molecular diagnostics—will be essential for improving maternal and neonatal outcomes in ICP.

## Figures and Tables

**Table 1:** Recommendations concerning time of delivery in intrahepatic cholestasis of pregnancy.

Recommendations Concerning Time of Delivery in Intrahepatic Cholestasis of Pregnancy		
Organization	Criteria	Recommended Time of Delivery
American College of Obstetricians and Gynecologists (ACOG)	Total bile acid levels < 100 micromol/L	36 0/7–39 0/7 or at diagnosis
	Total bile acid levels ≥ 100 micromol/L	36 0/7 or at diagnosis
	Delivery before 36 weeks of gestation occasionally	
Royal College of Obstetricians and Gynecologists (RCOG)	Peak bile acids 19–39 micromol/L	by 40 weeks’ gestation
	Peak bile acids 40–99 micromol/L	38–39 weeks’ gestation
	Peak bile acids 100 micromol/L	35–36 weeks’ gestation
International Federation of Gynecology and Obstetrics (FIGO)	Non-fasting serum bile acid level 10–39 micromol/L	37–39 weeks
	Non-fasting serum bile acid level ≥ 40–99 micromol/L	36–39 weeks (closer to 36)
	Non-fasting serum bile acid level ≥ 100 micromol/L	35–36 weeks

Refs. [52,70,71].

**Table 2:** Potential future directions in pharmacotherapy.

Therapy	Mechanism of Action	Evidence	Pregnancy Safety
IBAT inhibitors	↓ BA reabsorption in the ileum, ↑ fecal BA excretion	Phase II clinical trials in women with ICP	Likely safe; Gastrointestinal adverse effects
FXR agonists	↓ BA synthesis, ↑ hepatic bile acid transport via FXR activation	Phase III clinical trials in cholestatic liver disease	Not tested in pregnancy yet
Norursodeoxycholic acid (NorUDCA)	↑ Bile flow, ↓ liver inflammation and fibrosis	Phase II trials in primary sclerosing cholangitis	Not tested in pregnancy yet
4-Phenylbutyrate (4-PBA)	↑ BSEP expression, ↓ Endoplasmic reticulum stress and hepatocyte injury	Preclinical efficacy in murine cholestasis models	Preclinical only; no human pregnancy data
BACH1 inhibitors	↓ Placental oxidative stress, ↑ angiogenesis	Experimental animal models	Unknown; no human data
Probiotics	Modulation of gut microbiota and bile acid reabsorption	Pilot studies suggest symptom relief and bile acid improvement	Likely safe; well-tolerated in pregnancy

## Abbreviations

The following abbreviations are used in this manuscript:

ICP	Intrahepatic Cholestasis of Pregnancy
BA	Bile Acids
IBD	Inflammatory Bowel Disease
HBV	Hepatitis B virus
IVF	In Vitro Fertilization
BSEP	Bile Salt Export Pump
AST	Aspartate Aminotransferase
ALT	Alanine Transaminase
AP	Alkaline Phosphatase
GGT	Gamma-Glutamyl Transferase
UDCA	Ursodeoxycholic Acid
GDM	Gestational Diabetes Mellitus
CDCA	Chenodeoxycholic Acid
CA	Cholic Acid
LPA	The Lysophosphatidic acid
IBAT	Intestinal Bile Acid transporter
NorUDCA	Norursodeoxycholic Acid
E2	17α-Ethynylestradiol
OCA	Obeticholic Acid
SCFAs	Short-Chain Fatty Acids
BSH	Bile Salt Hydrolase
NLR	Neutrophil-To-Lymphocyte Ratio
SII	Immune-Inflammation Index
4-PBA	4-Phenylbutyric Acid
AUC	Area Under the Curve
aRR	Adjusted Risk Ratio
aOR	Adjusted Odds Ratio
CI	Confidence Interval
MD	Mean Deviation
